# The number of prior knee arthroscopies is associated with an incremental increase in risk of revision in a subsequent total knee arthroplasty

**DOI:** 10.1002/ksa.70038

**Published:** 2025-08-28

**Authors:** Julius Tetens Hald, Anders Odgaard, Michael Mørk Petersen, Anders El‐Galaly

**Affiliations:** ^1^ Department of Orthopedic Surgery Rigshospitalet, Copenhagen University Copenhagen Denmark; ^2^ Department of Clinical Medicine University of Copenhagen Copenhagen Denmark

**Keywords:** knee arthroplasty, knee arthroscopy, knee degeneration, risk of revision, survival analysis

## Abstract

**Purpose:**

The aim of this study was to estimate the relative risk of revision for total knee arthroplasty (TKA) with prior knee arthroplasties compared to TKAs in knees without prior surgery. In addition, this study aimed to assess if there was a dose‐response relationship between the number of prior knee arthroscopies and the risk of TKA revision.

**Methods:**

A retrospective observational study of three Danish Health Registries. All primary TKAs performed in Denmark from 1998 to 2021 were identified. Knee arthroscopies prior to primary arthroplasty were identified for these knees. The patients were grouped by whether they had an arthroscopy prior to the TKA or not. Kaplan–Meier analysis and Cox regression analysis was used to estimate implant survival and hazard ratio (HR) for revision.

**Results:**

The study included 96,781 primary TKAs without prior surgery (de novo TKA) and 15,042 primary TKAs that had had one or more arthroscopies as only prior surgery. After adjusting for age, sex, and Charlson Comorbidity Index (CCI) the HR for revision was 1.38 (95% confidence interval [CI] 1.29–1.47, *p* < 0.001) for knees with prior arthroscopy compared to knees without prior arthroscopy. After adjusting for age, sex, and CCI each additional arthroscopy increased the HR for revision of 1.27 (95% CI 1.21–1.33, *p* < 0.001) in a dose‐response manner.

**Conclusion:**

Previous knee arthroscopies increase the risk of revision following primary TKA. More importantly, each additional knee arthroscopy increased the risk by 27%. Although the exact mechanism behind the observation is unknown, this is important information for both surgeons and patients when considering treatment options for knees affected by degenerative conditions.

**Level of Evidence:**

Level III, retrospective comparative study.

**Abbreviations**
BMI: body mass index
CCI: Charlson Comorbidity Index
DCRS: Danish Civil Registration System
De novo TKA: TKA's due to idiopathic arthrosis without any prior knee surgery
DKAR: Danish Knee Arthroplasty Register
DNPR: Danish National Patient Register
HR: hazard ratio
ICD‐10: 10th version of the international statistical classification of diseases and related health problems of the World Health Organisation
IQR: interquartile range
KOA: knee osteoarthritis
SD: standard deviation
STROBE: strengthening the reporting of observational studies in epidemiology
TKA: total knee arthroplasty

## INTRODUCTION

The treatment of knee osteoarthritis (KOA) has historically involved arthroscopic lavage and debridement [13, 14]. However, after the findings of Mosely et al. [14] and subsequent studies [12, 25], arthroscopic lavage and debridement have largely been abandoned as a treatment for KOA [PMID: 37394226]. Yet, knee arthroscopy is still indicated for the treatment of several traumatic knee conditions and a systematic review has found that 2.6% of patients undergoing an arthroscopic procedure progress to knee arthroplasty annually [25].

The impact of undergoing knee arthroscopy, regardless of the initial indication, prior to knee arthroplasty has received little attention. Recent studies have found that patients with previous arthroscopy have an increased risk of worse patient‐reported outcomes compared to those without previous arthroscopy [2, 11], and that knees with previous arthroscopy exhibit worse 2‐year implant survival [7]. The mid to long‐term implant survival of TKAs following arthroscopy has not been assessed, nor has the impact of multiple arthroscopies on the risk of revision been investigated. The aims of this study were to fill these knowledge gaps by estimating the mid to long‐term implant survival of TKAs following arthroscopy, and to assess if the number of previous knee arthroscopies increase the risk of TKA revision in a dose‐response manner.

This study hypothesised (1) that prior arthroscopy negatively affects TKA survival, (2) that this negative effect is not restricted to only the first two post‐TKA years, and (3) that there is an association between the number of prior arthroscopies and the risk of TKA revision.

## METHODS

### Design and registries

This is an observational cohort study based on registry data of primary TKAs performed in Denmark from 1 January 1 1998 to 31 December 2021 [8]. The following registries were used: The Danish National Patient Register (DNPR), The Danish Civil Registration System (DCRS), and the Danish Knee Arthroplasty Register (DKAR). The DNPR holds data on all contacts within the Danish healthcare system [20]. These include but are not limited to admissions, surgical procedures, other treatments, and diagnoses. The DKAR is a clinical database created in 1997 [16], and due to mandatory registration DKAR captures more than 90% of all arthroplasty procedures. Information from DNPR and the DKAR were merged to obtain a complete cohort of all TKAs inserted in Denmark during the study period. The completeness of DKAR was 97% in 2021 [15]. The DCRS holds data regarding death and emigration for all citizens in Denmark [19]. Reliable, patient‐level linkage between registries was obtained by utilising the Danish Civil Registration number, which is uniquely assigned to all Danish residents at the time of birth or immigration. The study is presented in adherence to the STROBE‐statement [5].

### Study population

All primary TKAs conducted due to primary or secondary osteoarthritis (e.g., post‐traumatic osteoarthritis) during the study period were included. TKAs performed due to inflammatory arthritis, fresh fractures, and tumours were not included. Bilateral observations were included, and thus one patient could contribute with two knees. From the DNPR, all prior knee surgery, performed in the same period and on the same knee, were identified. TKAs with any major knee procedures other than arthroscopies, such as fracture surgery, removal of bone tumours, bone transplants, or osteotomies were excluded. From this cohort, the observations were divided into two groups. The intervention group was TKAs with one or more prior knee arthroscopies and the control group was TKAs without prior knee surgery, denoted as ‘de novo TKA’.

### Baseline characteristics

Baseline characteristics were determined relative to the time of TKA surgery and included sex, age, indication for TKA, number of previous arthroscopies, the indication for the arthroscopy, Charlson Comorbidity Index (CCI) [4], and the time from the latest arthroscopy. The CCI was calculated by obtaining all patient‐linked diagnoses from the DNPR [21]. If more than 1 arthroscopy were conducted, the indication of the first included arthroscopy was presented and included in the Cox regression. The indications of the arthroscopies were given as ICD‐10 diagnoses in the DNPR. Based on these, the arthroscopies were categorised into two groups, ‘degenerative’ and ‘traumatic’. The specific ICD‐10 codes belonging to each group can be found in Supporting Information: Appendix A1. All other indications were grouped as ‘other’ and were not subject to analysis.

### Revision

A revision was defined as removal, addition, or exchange of any part of the knee arthroplasty, as defined by the DKAR. If more than one indication was registered, a clinical hierarchy was used to ensure only one indication was evaluated for each revision [6]. This hierarchy places ‘infection’ at the top and the indications aseptic loosening, instability, and others below. No distinction was made between minor (secondary patellar resurfacing, liner exchange) and major (exchange of tibial or femoral component) revisions.

### Analyses

The arthroplasty survival was compared between the groups, both as crude estimate and after adjustment for sex, age, CCI, number of prior arthroscopies, and the indication of the first arthroscopy. In addition, the subpopulation of knees with prior arthroscopies was analysed to evaluate the time from the latest arthroscopy to primary TKA, as well as the indication of the first arthroscopy performed.

### Sensitivity analyses

Due to the long study period, the analysis was repeated after dividing the study period into two parts (by 2010) to depict whether temporal changes in arthroplasty treatment might have affected the results.

### Statistics

Descriptive statistics included medians and interquartile ranges (IQR) and means and standard deviations (SD) depending on the data distribution. Differences between groups were tested by Welch's two‐sample *t*‐test and Pearson's Chi‐Square test. Missing data was reported if present. In the analyses, missing data was omitted. The implant survival of primary procedures was estimated by Kaplan–Meier plots with 95% confidence intervals (95% CI) and differences between groups were tested using the log‐rank test. Death, emigration, or end of observation period resulted in censoring. The Cox regression was used to estimate the relative hazard ratios (HR) of the risk of revision, with stepwise adjustment for potential confounders. Schoenfeld residual plots were used to detect non‐proportional hazards. First, the Cox regression using previous arthroscopies as a categorical variable (yes/no) was performed to test for a significant difference in the risk of revision. Afterwards the Cox regression analysis was repeated, now using previous arthroscopies as a numerical variable. This was done to test for a dose–response relationship. Rstudio version 4.3.1 was used [18].

## RESULTS

### Study cohort

**Figure 1:**
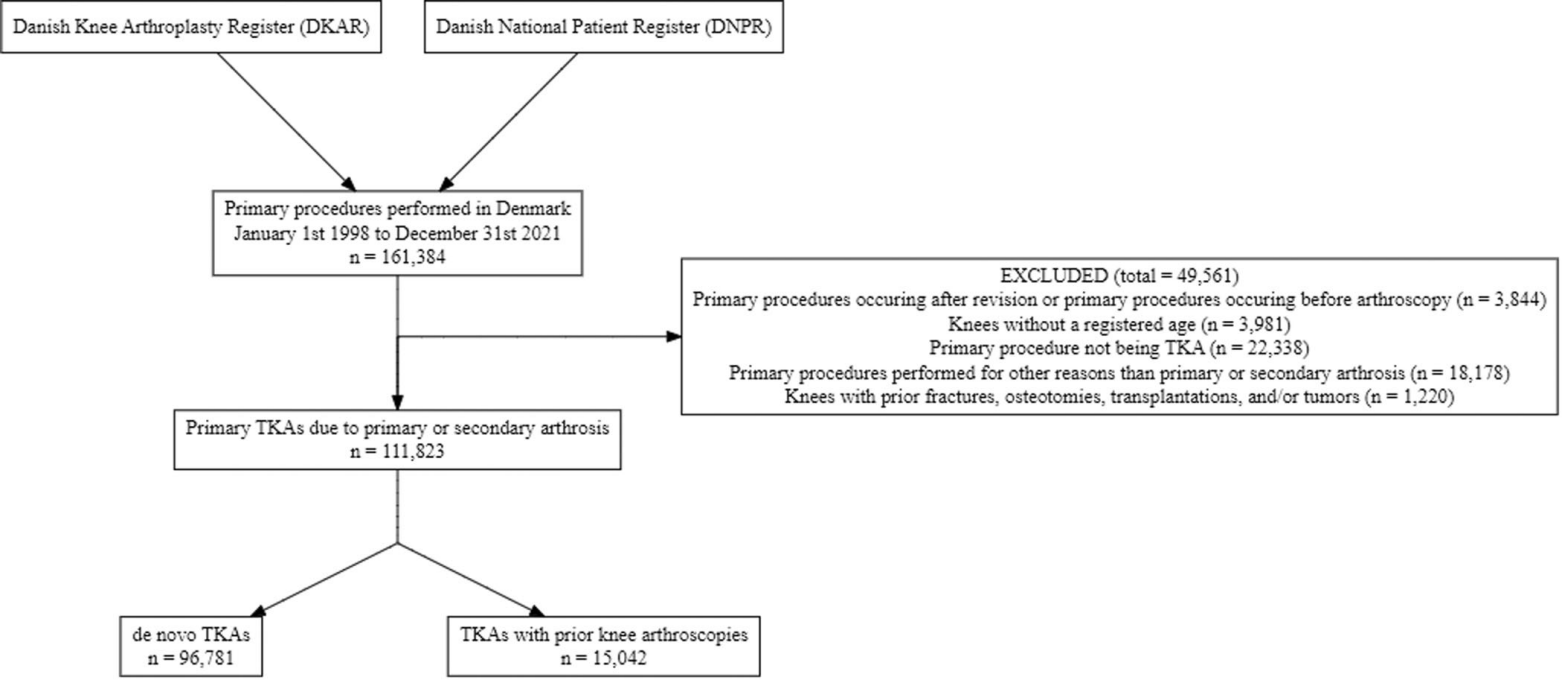
Flowchart of patient inclusion.

A total of 161,384 primary knee arthroplasties performed in Denmark from 1 January 1998 to 31 December 2021, were identified (Figure 1). In total 111,823 TKAs were included. Of these, 96,781 had no prior surgeries (de novo TKAs) and 15,042 TKAs had prior arthroscopy. 17,863 prior arthroscopies were identified, and 12,785 knees had had only one arthroscopy, while one knee had had seven arthroscopies. 11,471 arthroscopies were due to degenerative indications, 1946 arthroscopies were due to traumatic indications, and 1625 arthroscopies were due to other indications. No missing data concerning the indication for the arthroscopies was present. Baseline characteristics are available from Table 1.

**Table 1:** Baseline characteristics of knees undergoing primary TKA.

	De novo TKAs *n* = 96,781^a^	TKAs with 1 or more prior knee arthroscopies *n* = 15,042^a^	Difference [95% CI; *p*]^b^
Female sex	58,785 (61%)	8883 (59%)	<0.001
Age, years	70 (63–76)	63 (56–70)	6.6 [6.4–6.7; *p* < 0.001]
CCI			<0.001
0	60,061 (62%)	9428 (63%)	
1	16,732 (17%)	2803 (19%)	
2	10,953 (11%)	1528 (10%)	
>=3	9035 (9.3%)	1266 (8.4%)	
Missing	0	17 (0.1%)	
Time from latest arthroscopy to primary TKA, years	–	2.7 (0.7–7.4)	–

Abbreviations: CCI, Charlson Comorbidity Index; CI, confidence interval; TKA, total knee arthroplasty.

^a^ *n*/*N* (%); median (25% 75%).

^b^ Pearson's chi‐square test for categorical variables; Welch two sample *t*‐test for continuous variables.

### Implant survival and risk of TKA revision

**Figure 2:**
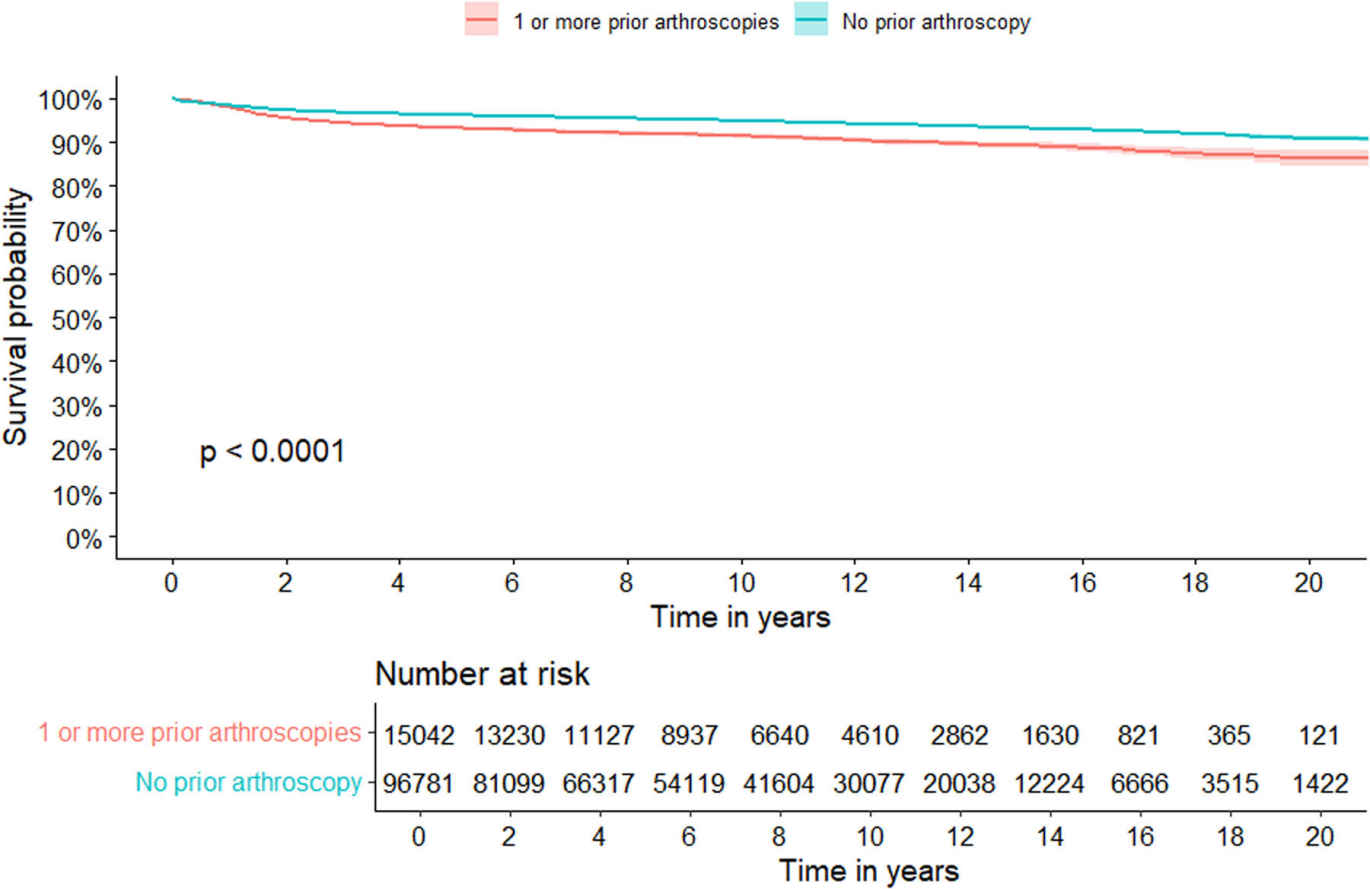
Kaplan–Meier analysis of the survival of primary knee arthroplasties.

The implant survival of de novo TKAs and the arthroscopy group are shown in Figure 2. The 20‐year implant survival probability of de novo TKAs was 91% [95% CI 90%–91%]. The 20‐year implant survival probability of knees with prior arthroscopy was 86% [95% CI 84%–88%], which corresponds to a significant difference between the groups (*p* < 0.001). Follow‐up was 0.15 years shorter for de novo TKAs compared to TKAs with 1 or more prior arthroscopy [95% CI 0.1–0.2].

The results of the Cox regression analysis are shown in Table 2. Without adjustment for covariates, it was found that knees with one or more prior arthroscopies had an increased risk of revision compared to de novo TKAs by a relative HR of 1.70 [95% CI 1.60–1.82, *p* < 0.001]. Following adjustment for sex, age, and CCI, it was found that knees with one or more prior arthroscopy had an increased risk of revision compared to de novo TKAs by a relative HR of 1.38 [95% CI 1.29–1.47, *p* < 0.001].

**Table 2:** Cox regression of arthroscopies, as a categorical variable, on the hazard of revision.

Values are given as hazard ratios [95% confidence interval]				
>=1 prior KA^a^	1.70 [1.60–1.82]	1.70 [1.60–1.82]	1.38 [1.29–1.48]	1.38 [1.29–1.47]
Male sex^b^		1.09 [1.03–1.15]	1.05 [1.00–1.11]	1.05 [0.99–1.11]
Age, years^c^			0.97 [0.97–0.97]	0.97 [0.96–0.97]
CCI–1^d^				1.19 [1.11–1.28]
CCI–2				1.19 [1.09–1.30]
CCI–>=3				1.48 [1.35–1.62]

*Note*: Increasing control for covariates.

^a^ Arthroscopies as a categorical value. 0 previous arthroscopies as reference.

^b^ Sex as a dichotomous variable. Female sex as reference.

^c^ Age as a continuous variable.

^d^ Charlson Comorbidity Index (CCI) as a categorical variable. 0 as reference.

When adjusting for the time from the latest arthroscopy to primary TKA the relative HR for revision also remained significant (HR 1.31 [95% CI 1.19–1.43, *p* < 0.001]).

### Arthroscopies – Dose–response relationship

Every numerical increase of previous arthroscopies increased the risk for revision by a relative adjusted HR of 1.27 [95% CI 1.21–1.33, *p* < 0.001] (Table 3). When stratified by the indication for the prior arthroscopy, arthroscopies performed due to degenerative indications increased the risk of TKA revision (relative HR 1.37 [95% CI 1.28–1.48, *p* < 0.001]) (Table 4). Arthroscopies performed due to trauma also increased the risk of TKA revision (relative HR 1.34 [95% CI 1.14–1.59, *p* < 0.001]).

**Table 3:** Cox regression of arthroscopies, as a continuous variable, on the hazard of revision.

Values are given as hazard ratios [95% confidence interval]				
Arthroscopies^a^	1.49 [1.43–1.55]	1.48 [1.42–1.55]	1.27 [1.22–1.33]	1.27 [1.21–1.33]
Male sex^b^		1.09 [1.03–1.15]	1.05 [1.00–1.11]	1.04 [0.99–1.10]
Age, years^c^			0.97 [0.97–0.97]	0.97 [0.96–0.97]
CCI–1^d^				1.19 [1.11–1.27]
CCI–2				1.19 [1.09–1.30]
CCI–>=3				1.47 [1.35–1.61]

*Note*: Increasing control for covariates.

^a^ Arthroscopies as a continuous variable.

^b^ Sex as a dichotomous variable. Female sex as reference.

^c^ Age as a continuous variable.

^d^ Charlson Comorbidity Index (CCI) as a categorical variable. 0 as reference.

**Table 4:** Cox regression of arthroscopies, grouped by the indication for the arthroscopy.

Values are given as hazard ratios [95% confidence interval]				
Arthroscopies due to degeneration^a^	1.68 [1.57–1.81]	1.68 [1.56–1.81]	1.38 [1.28–1.49]	1.37 [1.28–1.48]
Arthroscopies due to trauma	1.69 [1.43–2.0]	1.69 [1.43–1.99]	1.35 [1.14–1.59]	1.34 [1.14–1.59]
Male sex^b^		1.09 [1.03–1.15]	1.05 [1.00–1.11]	1.05 [0.99–1.10]
Age, years^c^			0.97 [0.97–0.97]	0.97 [0.96–0.97]
CCI–1^d^				1.19 [1.11–1.27]
CCI–2				1.19 [1.09–1.30]
CCI–>=3				1.48 [1.35–1.62]

*Note*: The number of prior arthroscopies is used as a continuous variable, on the relative hazard of revision. Increasing control for covariates.

^a^ No prior arthroscopy as reference.

^b^ Sex as a dichotomous variable. Female sex as reference.

^c^ Age as a continuous variable.

^d^ Charlson Comorbidity Index (CCI) as a categorical variable. 0 as reference.

### Indications for TKA revision

De novo TKAs, and the prior arthroscopy group differed in the overall proportion of the indications for the revisions (Table 5). The greatest differences in these proportions were revisions due to infection (13% higher for de novo TKAs [*p* < 0.001]), instability (6% lower for de novo TKAs [*p* < 0.001]) and pain (7% lower for de novo TKAs [*p* < 0.001]).

**Table 5:** Indications for revisions of primary TKAs grouped by prior knee arthroscopies.

	De novo TKAs *n* = 4311^a^	TKAs with 1 or more prior knee arthroscopies *n* = 1163^a^	Difference^b^ *p* < 0.001
Aseptic loosening	1,507 (35%)	416 (36%)	
Infection	1,166 (27%)	163 (14%)	
Instability	701 (16%)	259 (22%)	
Other	284 (6%)	56 (5%)	
Pain	260 (6%)	151 (13%)	
Secondary patella	236 (6%)	71 (6%)	
Wear	157 (4%)	47 (4%)	

Abbreviation: TKA, total knee arthroplasty.

^a^ *n*/*N* (%).

^b^ Pearson's chi‐square test.

### Sensitivity analysis

For both periods it was found that the number of previous arthroscopies increased the risk of revision: For the early group, after adjusting for age, sex and CCI, every increase in prior knee arthroscopies increased the HR for revision by a relative HR of 1.29 (95% CI 1.19–1.41, *p* < 0.001). For the late group, after adjusting for age, sex, and CCI, every increase in prior knee arthroscopies increased the relative HR for revision by 1.32 (95% CI 1.24–1.39, *p* < 0.001).

## DISCUSSION

The main finding of this study was that after adjusting for sex, age and CCI the risk of revision increased by a relative HR of 1.38 for knees with prior arthroscopy compared to de novo TKAs. The increased risk remained at 10 years and appeared to have a continuous effect. Using the number of prior arthroscopies as a continuous variable and after adjusting for sex, age and CCI, the risk of revision increased by a relative HR of 1.27 for each prior arthroscopy.

Previous studies have found that just one prior arthroscopy increases the risk of revision [2, 7]. The current study found similar implant survival probabilities as in the study of Piedade et al. [17]. Their study included 1119 de novo TKAs and 60 TKA patients with prior arthroscopic debridement. They found that patients with previous knee surgery had a primary TKA implant survival of 87% after 10 years while the 10‐year implant survival of de novo TKAs was 98%. In contrast, two other studies have found no difference in the implant survival between patients with prior arthroscopy and de novo TKAs [10, 23]. Both were smaller single‐centre studies and vulnerable to recall bias. The studies included 160 and 62 knees with prior arthroscopy, respectively. The implant survival of both groups was nearly identical.

The timing of the arthroscopy in relation to the insertion of the TKA also seems important. Two studies have shown that knees with arthroscopies performed less than 6 months prior to TKA have an increased risk of complications and revisions [1, 24]. This study found similar results; increasing time from the latest arthroscopy to the primary TKA reduced the risk of revision of an HR of 0.94. Thus, the finding supports the argument that surgeons should not perform TKA shortly after an arthroscopic procedure, although the HR decrease is rather modest. The indication of the first arthroscopy also significantly affected the risk of revision in subsequent TKA when compared to de novo TKA, however both traumatic and degenerative indications for arthroscopies equally increased the relative risk of revision in subsequent TKAs. This finding highlight that even ‘minor’ arthroscopies, such as house‐cleanings, are associated with decreased survival of subsequent TKAs.

The indication for revision of failed TKAs differed between the groups. Surprisingly, this study found fewer revisions due to infection in the arthroscopy group compared to the de novo TKA group. Two previous studies have reported higher incidences of revisions due to infection in the prior arthroscopy group [7, 24]. These studies focused on arthroscopies performed within 6 months of the primary TKA and thus time from arthroscopy to TKA might play a role in the risk of infection. Furthermore, this study highlighted that pain as an isolated indication for revision was more frequent in the prior arthroscopy group compared with de novo TKAs. The decision to perform a TKA due to ‘pain’ as the sole indication is controversial [22]. Furthermore, it is difficult to produce objective evidence in support of the indication ‘instability’ [9]. Both pain and instability can be considered as relative indications for TKA revision and thus, noteworthy, that these are more frequent in the prior arthroscopy group. One driver of this observation might be that patients, who have repeatedly been arthroscoped, may have higher requirements or present complaints in a more insisting manner that causes surgeons to suggest yet another operation to attempt improvement.

### Limitations

Some limitations are present within this study. Registries may contain missing or incomplete data which can affect the reliability of results. DKAR is known to have a lower completeness rate at the beginning of the observation period [16]. However, all medical procedures and admissions have been reported to the DNPR since the 1970's, which is required by Danish law [20] and a prerequisite for hospital payment. Combining information from DKAR and DNPR provided and almost complete dataset of all TKAs conducted during the study period. However, the high completeness limited the granularity of the data and thus, it was not possible to control for some confounding variables such as BMI and smoking. These are not available from the registries and may affect the results if they were unevenly distributed between the two groups. Patient income or wealth may influence access to healthcare. Denmark has a tax‐funded and available‐to‐all healthcare system with no out‐of‐pocket costs, so this effect is believed to be minimal. Changes in treatment protocols/regimens over the study period might also have affected the results of this study due to the long study period. However, the sensitivity analysis demonstrated that the clinical significance of this study's findings did not change between 1998–2010 versus 2010–2021. Last in this list of limitations is the fact that no distinction was made between minor and major revisions. A decision was made in the planning phase of this study not to focus on this distinction since the number of minor revisions is relatively low.

## CONCLUSION

This study found that after adjusting for age, sex and CCI, knees with prior arthroscopy had an increased risk of revision following primary TKA by an HR of 1.38 compared with de novo TKAs. The increased risk persisted beyond the first two years after TKA. This study found that every additional prior arthroscopy increased the risk of revision in subsequent TKAs by an HR of 1.27. This dose‐response relationship is especially important to consider when advising patients for another arthroscopy or for deciding the best timing of a TKA. This study has not identified a clear causal mechanism linking arthroscopy and subsequent TKA revision, but it is noted that TKAs with prior arthroscopies had a higher frequence of revision due to relative indications, pain and instability.

## AUTHOR CONTRIBUTIONS

All authors contributed to the study conception and design. Material preparation, data collection and analysis were performed by Julius Tetens Hald and Anders El‐Galaly. Anders Odgaard and Michael Mørk Petersen contributed significantly to conceptualisation, methodology, validation of results, funding acquisition, and supervision. The first draft of the manuscript was written by Julius Tetens Hald and all authors commented on previous versions of the manuscript. All authors read and approved the final manuscript.

## CONFLICT OF INTEREST STATEMENT

The authors declare no conflicts of interest.

## ETHICS STATEMENT

The study has been approved by the Danish Data Protection Agency which is governed by the Capital Region of Denmark (case number: P‐2022‐711). Register studies in Denmark are not required to obtain approval from the regional ethical committee.

## Supporting information

Supplementary Table 1: ICD‐10 codes listed in the registers and used for identifying the indication for the first arthroscopic procedure for each patient.

## Data Availability

The data that support the findings of this study are available from Statistics Denmark and The Danish Knee Arthroplasty Register. Restrictions apply to the availability of these data, which were used under license for this study. Data are available from the registers with the permission of The Danish Data Protection Agency.
