# Supplementary material for: The number of prior knee arthroscopies is associated with an incremental increase in risk of revision in a subsequent total knee arthroplasty
Source: Knee Surg Sports Traumatol Arthrosc. 2025 Aug 28;34(4):1327–35. doi: 10.1002/ksa.70038 (PMC13037344; doi:10.1002/ksa.70038)
Supplement: Supplementary file 1 — Supplementary Table 1: ICD‐10 codes listed in the registers and used for identifying the indication for the first arthroscopic procedure for each patient. [file KSA-34-1327-s001.docx]

| ICD-10 codes given as the indication for the first arthroscopic procedure | |
| --- | --- |
| Group = ’traumatic” | DM220  DM221  DM223  DM235  DM236  DM236A  DM244  DM252  DM664  DM999  DS761  DS800  DS801  DS810  DS820  DS821  DS822  DS823  DS824  DS828  DS830  DS831  DS832  DS832A  DS832B  DS834  DS834A  DS834B  DS835  DS835A  DS835B  DS835C  DS835D  DS835E  DS836  DS837  DS837C  DS862  DS899  DT930  DT933  DT933A  DT935  DT935B  DT938  DT939 |
| Group = ’Degenerative’ | DM139  DM148  DM150  DM154  DM170  DM171  DM171A  DM171B  DM172  DM173  DM173B  DM174  DM175  DM175A  DM175B  DM179  DM190  DM190A  DM191  DM192  DM199  DM224  DM229  DM230  DM231  DM232  DM233  DM233B  DM234  DM240  DM942 |
